# Correlation between tear cytokine profiles and corneal injury degree in patients with dry eye syndrome

**DOI:** 10.3389/fimmu.2026.1756076

**Published:** 2026-03-19

**Authors:** Lei Cao, Cuiyu Wang, Kai Zhang, Zhida You, Yingxin Chen

**Affiliations:** Department of Ophthalmology, General Hospital of Northern Theater Command, Shenyang, Liaoning, China

**Keywords:** corneal injury, dry eye syndrome, inflammatory factors, predict, tear cytokines

## Abstract

**Background:**

Dry eye syndrome (DES) is a very common ocular surface disorder that is closely related to corneal damage. However, the relationship between tear cytokines in patients with DES and the severity of corneal damage remains unclear.

**Methods:**

Patients with DES of different corneal injury degrees (mild, moderate, severe) were enrolled. The baseline data of each group of patients were compared. The levels of tears cytokines were detected by the multiplex Luminex assay. The Kruskal-Wallis H test and the Mann-Whitney U test were used to compare the levels of cytokines. Independent risk factors were screened out using the logistic regression method, and their predictive effects were evaluated by the ROC curve analysis. Ordered logistic regression analysis confirmed the association between key cytokines and the severity of corneal injury.

**Results:**

There were no significant differences in baseline data among the three groups (all *P* > 0.05). IL-1β, IL-6, IL-8, and TNF-α were identified as independent risk factors for moderate corneal injury, with the combined model achieving an AUC of 0.865. IL-1β, IL-6, and IL-8 were independent risk factors for severe corneal injury, and the combined model had an AUC of 0.885. The ordered logistic regression analysis further confirmed that IL-1β, IL-6, TNF-α and IL-8 were independent risk factors for the aggravation of corneal injury severity across all grades.

**Conclusion:**

IL-1β, IL-6, TNF-α and IL-8 play key roles in injury progression. The targeted combined models can efficiently predict the progression of corneal injury degree, providing reliable evidence for clinical graded management and individualized intervention.

## Introduction

Dry eye syndrome (DES) is one of the most common chronic diseases in ophthalmology (1). With the aging of the population and the widespread use of electronic devices, as well as changes in environmental factors, its incidence rate is increasing year by year (2, 3). DES is mainly characterized by reduced tear film stability, persistent ocular surface inflammation, and impairment of corneal structure and function (4). Patients often present with symptoms including dry eye, foreign body sensation, burning, stinging, and photophobia (5–7). The cornea, as the core carrier for maintaining the functions of the ocular surface, has become one of the main organs affected by DES pathological damage (8). As the most anterior transparent tissue of the ocular surface, the cornea not only plays a crucial role in light refraction, but also fulfills important functions such as barrier protection and sensory transmission (9). The occurrence and development of corneal injury are closely related to the imbalance of the ocular surface microenvironment (10). In particular, the abnormal regulation of the tear cytokine network may be the key link connecting ocular surface inflammation with corneal structural damage (11, 12). At present, there are still deficiencies in the analysis of the correlation between key tear cytokines in DES patients and the severity of corneal damage.

Tears are an important component of the ocular surface microenvironment. The cytokines present in tears play a crucial role in regulating ocular surface inflammatory responses and immune homeostasis (13). Current research indicates that the levels of cytokines (such as IL-1β, IL-2, IL-4, IL-6, IL-8, IL-10, IL-17A, IFN-γ, TNF-α, MMP-9 and VEGF) in patients with DES are significantly higher than those in healthy individuals (14–16). Among them, Yang et al. discovered that the increase in IL-1β levels in tears is associated with delayed corneal wound healing (17). Sugioka et al. demonstrated that IL-1β can promote the degradation of extracellular collagen by corneal fibroblasts (18). Additionally, Ebihara et al. confirmed that IL-6 can effectively stimulate corneal epithelial cell migration and promote corneal injury repair in both *in vitro* and *in vivo* settings (19, 20). However, the correlation between cytokines in tears and the severity of corneal injury has not been clearly established, which significantly limits the ability to accurately assess and intervene in the risk of corneal injury progression in clinical practice.

Based on this, we examined the levels of cytokines in the tear fluid of DES patients with different degrees of corneal injury. It deeply analyzed the correlation between the cytokine profile and corneal injury degree, screened out the core factors driving the progression of the injury and constructed a combined prediction model. This provides more targeted experimental evidence for the grading diagnosis, risk warning, and individualized treatment of corneal injury in DES patients.

## Materials and methods

### Research subjects and grouping

A total of 164 patients diagnosed with DES in the ophthalmology outpatient department of our hospital between October 2021 and November 2023 were enrolled as the case group. The case group was divided into mild, moderate, and severe groups based on the degree of corneal injury. The stratification criteria were based on the corneal fluorescein staining (FL) score. The FL scoring system employs an improved four-quadrant grading system, dividing the cornea into four quadrants: superior, inferior, nasal, and temporal (excluding the central cornea). The scoring range for each quadrant is from 0 to 3: 0 = no staining; 1 = 1–30 punctate staining; 2 = >30 punctate staining but not fused; 3 = fused staining, filamentous substances or ulcers. The total FL score ranges from 0 to 12 (the sum of the four quadrants) (21, 22). The mild injury group: FL score 1-3, mild punctate staining of the corneal epithelium; the moderate injury group: FL score 4-8, multiple punctate or patchy staining of the corneal epithelium, without involving the central corneal area; the severe injury group: FL score 9-12, extensive staining of the corneal epithelium or involving the central area, accompanied by epithelial defect. FL was performed using sodium fluorescein ophthalmic test strips (Tianjin Jingming Company) and evaluated under cobalt blue light of a slit-lamp microscope.

Inclusion criteria: (1) Meet the diagnostic criteria of “Chinese Expert Consensus on Clinical Diagnosis and Treatment of Dry Eye (2024) (23)”; (2) Age 18–65 years old, gender not limited; (3) Voluntarily participate in this study and sign the informed consent form. Exclusion criteria: (1) Comorbid other ocular diseases (such as keratitis, glaucoma, cataract) or refractive surgery history; (2) Systemic immune diseases (such as rheumatoid arthritis, Sjögren’s syndrome), diabetes mellitus, or other systemic disorders; (3) patients who used glucocorticoids, immunomodulators or artificial tears within 1 month pre-enrollment (excluding case group patients with standardized treatment); (4) Pregnant or lactating females; (5) Unsuccessful tear sample collection or non-cooperation with study procedures. This research has been approved by the ethics committee.

### Baseline data collection

All baseline information was collected including age, gender, BMI, smoking history, and drinking history. These data were obtained through questionnaire surveys and medical record reviews.

### Tear fluid collection

Tear samples were collected using the sterile capillary method without surface anesthesia and without stimulation during the operation. Due to the reduced basal tear secretion in dry eye patients, samples were collected from both eyes and pooled to obtain sufficient volume. For each sample, approximately 25-50 μL of tear fluid was collected in total (approximately 10-25 μL per eye). Samples were immediately frozen at -80°C for subsequent use.

### Tear cytokine detection the cytokine magnetic bead panel

The cytokines (IL-1β, IL-2, IL-6, IL-8, IL-17A, TNF-α, IFN-γ, IL-4, IL-10, TGF-β1, VEGF) in the tear fluid were simultaneously quantified using a multiplex Luminex assay kit (ProcartaPlex™ Human Cytokine Panel, Thermo Fisher). The assay was performed strictly following the manufacturer’s optimized protocol. In brief, 25 μL of tear sample was incubated with antibody-conjugated magnetic microbeads for 2 hours at room temperature, followed by incubation with biotinylated detection antibodies and streptavidin-PE for signal amplification. Fluorescence intensity was measured using a Luminex^®^ 200™ analyzer, which identified each cytokine by microsphere color code and quantified its concentration by PE fluorescence intensity. All samples were tested three times. The cytokine concentrations were calculated using the standard curves generated with the ProcartaPlex Analyst software. The detection sensitivity ranged from 1.2 to 4.0 pg/mL for the analytes.

### Data statistics and analysis

Statistical analysis was conducted using SPSS 26.0 (IBM, USA), GraphPad Prism 9.0 (GraphPad, USA) software, and R software (version 4.3.1; R Foundation for Statistical Computing, Vienna, Austria). Categorical variables were expressed as frequencies (n), and comparisons among the three groups were performed using the chi-square test. Continuous variables were first subjected to normality testing using the Shapiro-Wilk test. For variables that followed a normal distribution, data were presented as mean ± standard deviation (Mean ± SD), and comparisons among the three groups were conducted using one-way ANOVA. For non-normally distributed variables, data were expressed by the median and the interquartile range (IQR); overall comparisons among the three groups were performed using the Kruskal-Wallis H test, and pairwise comparisons (mild group vs. moderate group, moderate group vs. severe group) were conducted using the Mann-Whitney U test. Binary logistic regression analysis was used to separately identify independent risk factors for progression from mild to moderate and from moderate to severe corneal damage. Corneal damage severity (mild = 1, moderate = 2, severe = 3) was defined as an ordinal dependent variable, and the Brant test was used to verify the proportional odds assumption. If the assumption was valid, a proportional odds ordinal logistic regression model was applied; otherwise, a generalized ordinal logistic regression model was used. The diagnostic value of independent risk factors and combined models is analyzed by receiver operating characteristic (ROC) curve. The accuracy of the model was tested using the Bootstrap method. Values below the lower limit of detection (LLD) were replaced with LLD/2; samples above the upper limit of quantification (ULQ) were diluted and re-measured, and final concentrations were adjusted according to the dilution factor. A difference is considered statistically significant if *P* < 0.05.

## Results

### Baseline data of the research subjects

The baseline data analysis results of the research subjects with different degrees of corneal injury (mild, moderate, severe) are shown in **Table 1**. There were no statistically significant differences among the groups in terms of gender, age, BMI, smoking history, and alcohol consumption history (all *P* > 0.05).

**Table 1:** Baseline data of the research subjects.

Characteristics		Mild	Moderate	Severe	*P* value
		(*n* = 49)	(*n* = 41)	(*n* = 34)	
Gender	Male (n)	23	23	15	0.540
	Female (n)	26	18	19	
Age (Years)		37.37 ± 7.41	36.46 ± 8.20	38.11 ± 6.58	0.631
BMI (kg/m^2^)		23.86 ± 4.00	23.63 ± 4.17	23.82 ± 3.62	0.958
Smoking (Yes, n)		27	20	13	0.318
Drinking (Yes, n)		25	22	17	0.946

### Overall comparison of tear cytokine levels among the three groups

The Kruskal-Wallis H test was used to conduct a comprehensive comparison of the cytokine levels among the mild, moderate, and severe groups. The results showed that the levels of cytokines including IL-1β, IL-2, IL-6, IL-8, TNF-α, IL-17A, IFN-γ, and MMP-9 gradually increased with the severity of corneal injury (all *P* < 0.001). In contrast, the levels of IL-4, IL-10, TGF-β1, and VEGF showed no significant overall differences among the three groups (all *P* > 0.05) (**Table 2**).

**Table 2:** Comparison of cytokine levels among the three groups of patients.

Tear cytokines	Mild	Moderate	Severe	*P* value
IL-1β (pg/mg)	18.50 (8.57)	27.69 (13.7)	34.61 (13.77)	<0.001
IL-2 (pg/mg)	36.09 (3.71)	35.74 (3.09)	62.66 (9.82)	<0.001
IL-4 (pg/mg)	37.74 (16.26)	37.56 (14.49)	35.77 (10.36)	0.701
IL-6 (pg/mg)	19.35 (8.42)	24.91 (10.74)	46.54 (5.57)	<0.001
IL-8 (pg/mg)	26.44 (17.33)	39.06 (14.31)	95.95 (28.13)	<0.001
IL-10 (pg/mg)	46.59 (19.50)	45.58 (19.47)	44.27 (25.10)	0.850
TNF-α (pg/mg)	11.35 (8.42)	16.15 (5.80)	31.62 (4.25)	<0.001
IL-17A (pg/mg)	5.79 (0.35)	36.51 (5.17)	55.58 (7.81)	0.000
IFN-γ (pg/mg)	19.97 (6.12)	49.63 (4.78)	119.10 (6.56)	0.000
TGF-β1 (pg/mg)	96.59 (17.23)	98.76 (17.46)	97.93 (17.57)	0.655
VEGF (pg/mg)	67.81 (11.81)	65.96 (9.35)	65.55 (8.19)	0.231
MMP-9 (pg/mg)	17.38 (6.12)	33.05 (4.10)	56.72 (5.68)	0.000

IL-1β, interleukin-1 beta; IL-2, interleukin-2; IL-4, interleukin-4; IL-6, interleukin-6; IL-8, interleukin-8; IL-10, interleukin-10; TNF-α, tumor necrosis factor-alpha; IL-17A, interleukin-17A; IFN-γ, interferon-gamma; TGF-β1, transforming growth factor-beta 1; VEGF, vascular endothelial growth factor; MMP-9, matrix metalloproteinase-9.

### Tear cytokine levels and predictive efficacy: mild vs. moderate groups

The levels of tear cytokines were compared between the mild group and the moderate group using the Mann-Whitney U test. As shown in **Table 3**, the levels of IL-1β, IL-6, IL-8, TNF-α, IL-17A, IFN-γ and MMP-9 in the tear of the moderate group were significantly higher than those in the mild group. In contrast, there were no significant differences in the levels of IL-2, IL-4, IL-10, TGF-β1, and VEGF between the two groups.

**Table 3:** Comparison of tear cytokine levels between the mild group and the moderate group.

Tear cytokines	Mild	Moderate	*P* value
IL-1β (pg/mg)	18.50 (8.57)	27.69 (13.7)	<0.001
IL-2 (pg/mg)	36.09 (3.71)	35.74 (3.09)	0.478
IL-4 (pg/mg)	37.74 (16.26)	37.56 (14.49)	0.811
IL-6 (pg/mg)	19.35 (8.42)	24.91 (10.74)	<0.001
IL-8 (pg/mg)	26.44 (17.33)	39.06 (14.31)	<0.001
IL-10 (pg/mg)	46.59 (19.50)	45.58 (19.47)	0.473
TNF-α (pg/mg)	11.35 (8.42)	16.15 (5.80)	<0.001
IL-17A (pg/mg)	5.79 (0.35)	36.51 (5.17)	<0.001
IFN-γ (pg/mg)	19.97 (6.12)	49.63 (4.78)	<0.001
TGF-β1 (pg/mg)	96.59 (17.23)	98.76 (17.46)	0.913
VEGF (pg/mg)	67.81 (11.81)	65.96 (9.35)	0.530
MMP-9 (pg/mg)	17.38 (6.12)	33.05 (4.10)	<0.001

IL-1β, interleukin-1 beta; IL-2, interleukin-2; IL-4, interleukin-4; IL-6, interleukin-6; IL-8, interleukin-8; IL-10, interleukin-10; TNF-α, tumor necrosis factor-alpha; IL-17A, interleukin-17A; IFN-γ, interferon-gamma; TGF-β1, transforming growth factor-beta 1; VEGF, vascular endothelial growth factor; MMP-9, matrix metalloproteinase-9.

We performed univariate and multivariate logistic regression analyses to further explore the potential relationship between these differentially expressed cytokines and the progression from mild to moderate corneal injury. The results are shown in **Table 4**. In the univariate analysis, IL-1β, IL-6, IL-8, TNF-α, IL-17A, IFN-γ and MMP-9 were identified as factors related to the development from mild to moderate corneal injury. The multivariate analysis included variables that had *P* < 0.05 in the univariate analysis. IL-1β (OR = 6.645, 95% CI: 2.079 - 21.355, *P* = 0.001), IL-6 (OR = 3.488, 95% CI: 1.128 - 10.785, *P* = 0.030), IL-8 (OR = 5.127, 95% CI: 1.619 - 16.240, *P* = 0.005) and TNF-α (OR = 3.280, 95% CI: 1.032 - 10.424, *P* = 0.044) were independent risk factors for moderate corneal injury.

**Table 4:** Results of logistic regression analysis for the mild group and the moderate group .

Tear cytokines	Univariate analysis			Multivariate analysis		
	OR	95% CI	*P*	OR	95% CI	*P*
IL-1β	6.641	2.632-16.757	<0.001	6.645	2.079-21.355	0.001
IL-2	1.077	0.469-2.470	0.862	–	–	–
IL-4	0.648	0.281-1.493	0.308	–	–	–
IL-6	2.737	1.162-6.447	0.021	3.488	1.128-10.785	0.030
IL-8	3.709	1.542-8.921	0.003	5.127	1.619-16.240	0.005
IL-10	1.135	0.490-2.628	0.768	–	–	–
TNF-α	3.321	1.394-7.915	0.007	3.280	1.032-10.424	0.044
IL-17A	2.467	1.054-5.777	0.038	2.009	0.636-6.344	0.234
IFN-γ	2.513	1.071-5.901	0.034	2.568	0.805-8.196	0.111
TGF-β1	1.150	0.497-2.660	0.743	–	–	–
VEGF	0.470	0.201-1.099	0.081	–	–	–
MMP-9	2.872	1.206-6.839	0.017	2.193	0.718-6.695	0.168

IL-1β, interleukin-1 beta; IL-2, interleukin-2; IL-4, interleukin-4; IL-6, interleukin-6; IL-8, interleukin-8; IL-10, interleukin-10; TNF-α, tumor necrosis factor-alpha; IL-17A, interleukin-17A; IFN-γ, interferon-gamma; TGF-β1, transforming growth factor-beta 1; VEGF, vascular endothelial growth factor; MMP-9, matrix metalloproteinase-9.

**Figure 1:**
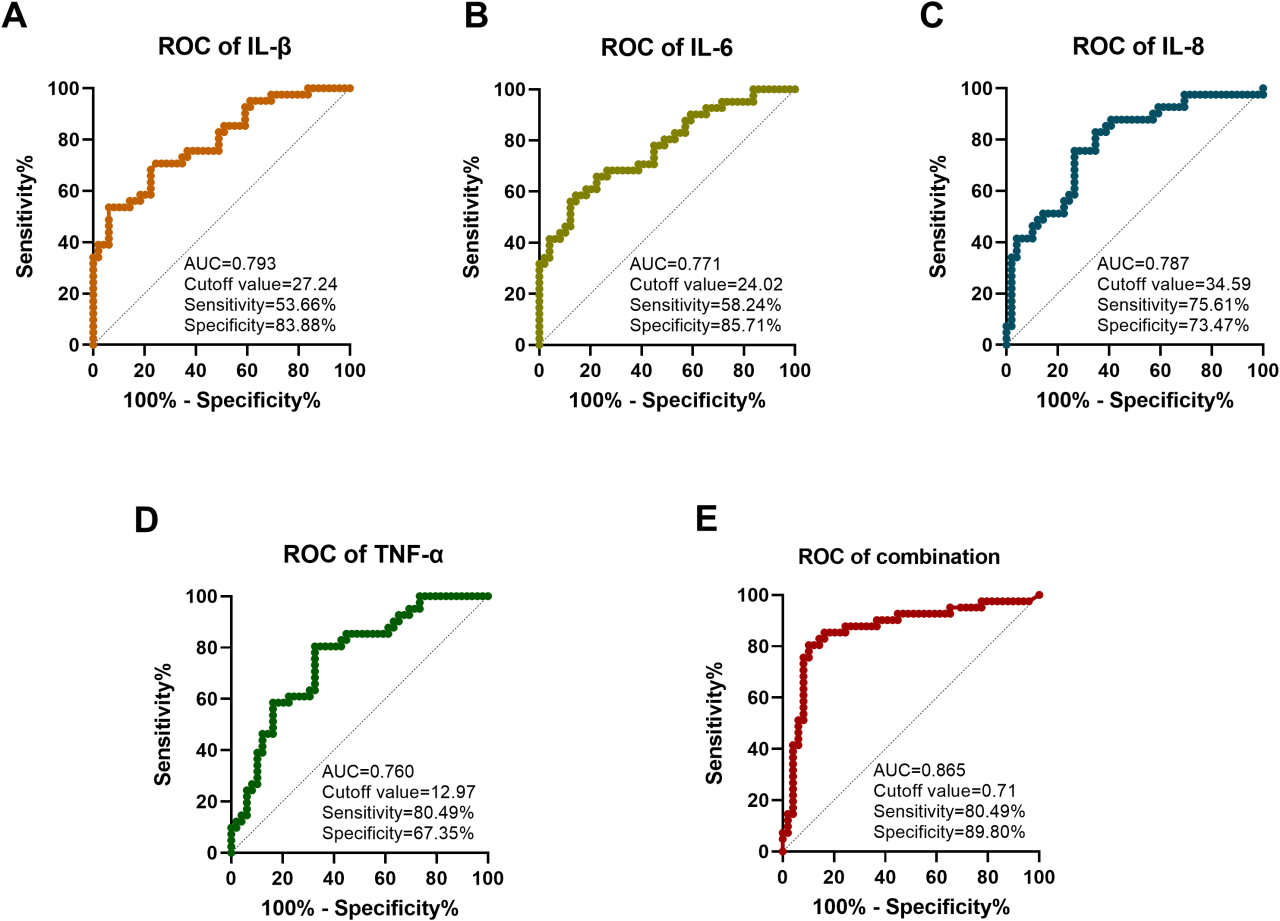
Analysis of the diagnostic efficacy of cytokines in the ROC curve for moderate corneal injury. **(A)** ROC curve analysis of IL-β. **(B)** ROC curve analysis of IL-6. **(C)** ROC curve analysis of IL-8. **(D)** ROC curve analysis of TNF-α. **(E)** ROC curve analysis of the multi-factor combined model.

The diagnostic efficacy of these independent risk factors in predicting the progression from mild corneal injury to moderate corneal injury using ROC curve analysis, as shown in **Figure 1**. The results indicated that the AUC of IL-1β was 0.793, with a cutoff value of 27.24, a sensitivity of 53.66%, and a specificity of 83.88%; the AUC of IL-6 was 0.771, with a cutoff value of 24.02, a sensitivity of 58.24%, and a specificity of 85.71%; the AUC of IL-8 was 0.787, with a cutoff value of 34.59, a sensitivity of 75.61%, and a specificity of 73.47%; the AUC of TNF-α was 0.760, with a cutoff value of 12.97, a sensitivity of 80.49%, and a specificity of 67.35%. It is noteworthy that the combined model of these four independent cytokines (IL-1β, IL-6, IL-8, and TNF-α) showed higher diagnostic efficacy, with an AUC of 0.865, a cutoff value of 0.71, a sensitivity of 80.49%, and a specificity of 89.80%. After 1000 Bootstrap resampling validations, the model’s concordance index (C-index, equivalent to AUC) was 0.727, confirming the stability and reliability of its predictive performance.

### Tear cytokine levels and predictive efficacy: moderate vs. severe groups

This study compared the cytokines in the tears of moderate and severe patients using the Mann-Whitney U test. The results are shown in **Table 5**. The levels of IL-2, IL-17A, IFN-γ, IL-1β, IL-6, IL-8, TNF-α and MMP-9 in the tears of the severe group were significantly higher than those of the moderate group. In contrast, there was no statistically significant difference such as IL-4, IL-10, TGF-β1, and VEGF between the two groups (*P* > 0.05).

**Table 5:** Comparison of tear cytokine levels between the moderate group and the severe group.

Tear cytokines	Moderate	Severe	*P* value
IL-1β (pg/mg)	27.69 (13.7)	34.61 (13.77)	0.010
IL-2 (pg/mg)	35.74 (3.09)	62.66 (9.82)	<0.001
IL-4 (pg/mg)	37.56 (14.49)	35.77 (10.36)	0.264
IL-6 (pg/mg)	24.91 (10.74)	46.54 (5.57)	<0.001
IL-8 (pg/mg)	39.06 (14.31)	95.95 (28.13)	<0.001
IL-10 (pg/mg)	45.58 (19.47)	44.27 (25.10)	0.856
TNF-α (pg/mg)	16.15 (5.80)	31.62 (4.25)	<0.001
IL-17A (pg/mg)	36.51 (5.17)	55.58 (7.81)	<0.001
IFN-γ (pg/mg)	49.63 (4.78)	119.1 (6.56)	<0.001
TGF-β1 (pg/mg)	98.76 (17.46)	97.93 (17.57)	0.856
VEGF (pg/mg)	65.96 (9.35)	65.55 (8.19)	0.941
MMP-9 (pg/mg)	33.05 (4.10)	56.72 (5.68)	<0.001

IL-1β, interleukin-1 beta; IL-2, interleukin-2; IL-4, interleukin-4; IL-6, interleukin-6; IL-8, interleukin-8; IL-10, interleukin-10; TNF-α, tumor necrosis factor-alpha; IL-17A, interleukin-17A; IFN-γ, interferon-gamma; TGF-β1, transforming growth factor-beta 1; VEGF, vascular endothelial growth factor; MMP-9, matrix metalloproteinase-9.

To clarify the independent predictive value of tear cytokines for severe corneal injury, this study used a logistic regression model to conduct a systematic evaluation of the cytokines. The univariate analysis showed that IL-1β, IL-6, IL-8, TNF-α, and MMP-9 were significantly associated with severe corneal injury (**Table 6**, all *P* < 0.05). By including the indicators with *P* < 0.05 from the univariate analysis in the multivariate analysis, the results showed that IL-1β (OR = 4.928, 95% CI: 1.525–15.923, *P* = 0.008), IL-6 (OR = 4.302, 95% CI: 1.313–14.097, *P* = 0.016), and IL-8 (OR = 4.373, 95% CI: 1.288–14.846, *P* = 0.018) were independent risk factors for severe corneal injury.

**Table 6:** Results of logistic regression analysis for the moderate group and the severe group.

Tear cytokines	Univariate analysis			Multivariate analysis		
	OR	95% CI	*P*	OR	95% CI	*P*
IL-1β	4.629	1.737-12.336	0.002	4.928	1.525-15.923	0.008
IL-2	1.619	0.648-4.044	0.303	**-**	**-**	**-**
IL-4	1.303	0.524-3.240	0.570	**-**	**-**	**-**
IL-6	3.624	1.389-9.458	0.009	4.302	1.313-14.097	0.016
IL-8	3.178	1.232-8.200	0.017	4.373	1.288-14.846	0.018
IL-10	0.847	0.341-2.104	0.720	**-**	**-**	**-**
TNF-α	2.865	1.116-7.352	0.029	2.456	0.784-7.691	0.123
IL-17A	0.847	0.341-2.104	0.720	**-**	**-**	**-**
IFN-γ	1.979	0.786-4.981	0.147	**-**	**-**	**-**
TGF-β1	1.467	0.588-3.658	0.411	**-**	**-**	**-**
VEGF	1.071	0.431-2.662	0.882	**-**	**-**	**-**
MMP-9	2.800	1.095-7.163	0.032	3.458	1.022-11.701	0.046

IL-1β, interleukin-1 beta; IL-2, interleukin-2; IL-4, interleukin-4; IL-6, interleukin-6; IL-8, interleukin-8; IL-10, interleukin-10; TNF-α, tumor necrosis factor-alpha; IL-17A, interleukin-17A; IFN-γ, interferon-gamma; TGF-β1, transforming growth factor-beta 1; VEGF, vascular endothelial growth factor; MMP-9, matrix metalloproteinase-9.

**Figure 2:**
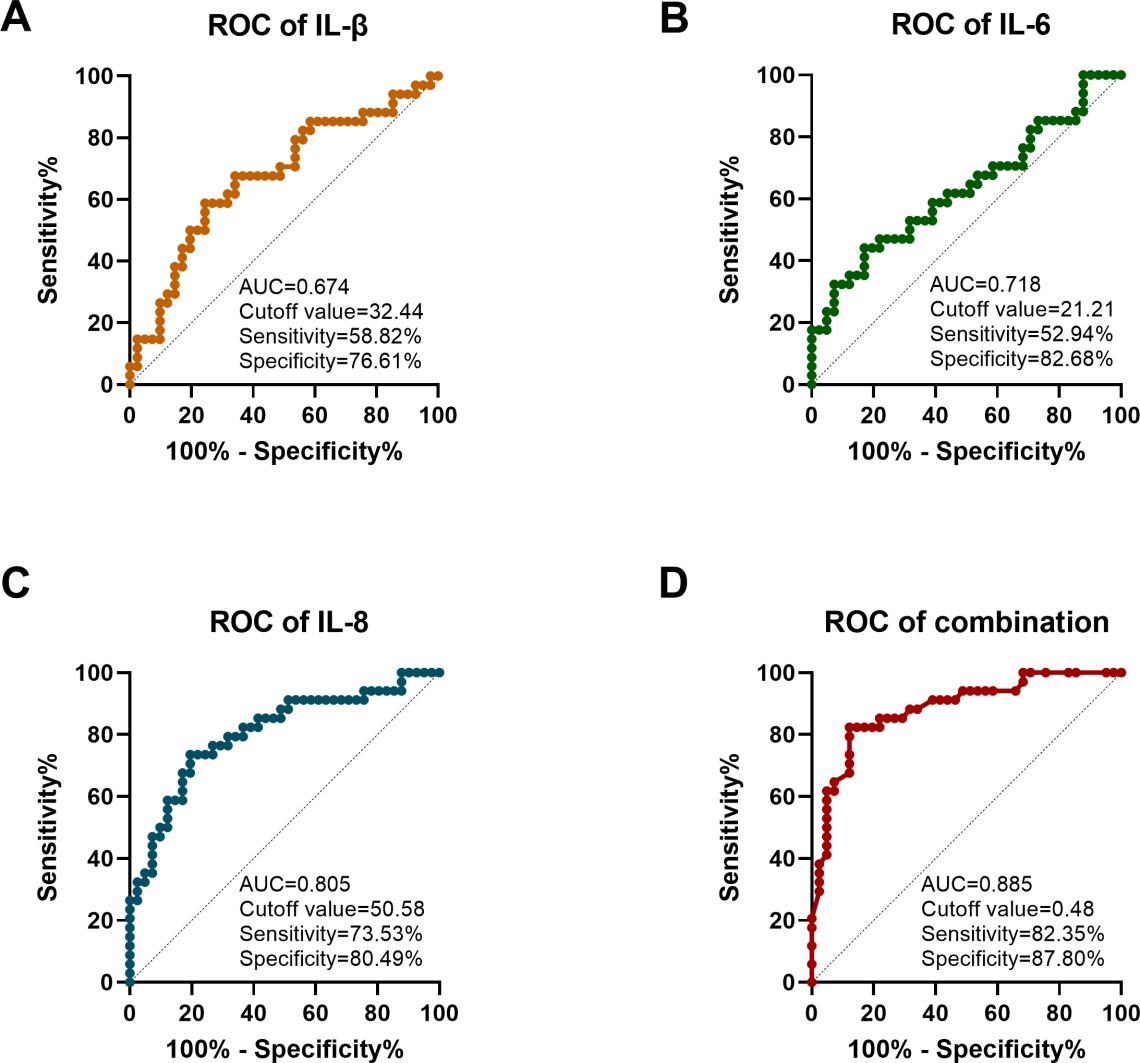
Analysis of the diagnostic efficacy of cytokines in the ROC curve for severe corneal injury. **(A)** ROC curve analysis of IL-β. **(B)** ROC curve analysis of IL-6. **(C)** ROC curve analysis of IL-8. **(D)** ROC curve analysis of the multi-factor combined model.

This study assessed the effectiveness of each independent risk factor and their combined prediction for severe corneal injury using the ROC curve based on the independent risk factors found through multivariate logistic regression. As shown in **Figure 2**, the AUC of IL-1β was 0.674, with a sensitivity of 58.82% and a specificity of 76.61% when the cutoff value was 32.44; the AUC of IL-6 was 0.718, with a cutoff value of 21.21, a sensitivity of 52.94% and a specificity of 82.68%; the AUC of IL-8 reached 0.805, with a cutoff value of 50.58, sensitivity 73.53%, specificity 80.49%. The AUC for differentiating moderate from severe DES using the combined index was 0.885, and when the cutoff value was 0.48, the sensitivity was up to 82.35% and the specificity was 87.80%. After 1000 Bootstrap resampling validations, the model’s C-index was 0.768 indicating that the model has strong discriminatory ability and stable results.

### Ordinal logistic regression analysis of tear cytokines and corneal injury severity

We set the severity of corneal injury (mild = 1, moderate = 2, severe = 3) as an ordinal dependent variable and used the parallel line test to evaluate the proportionality assumption, obtaining a chi-square value of 0.337 (degrees of freedom = 5, *P* = 0.997), indicating that the assumption was met and the standard ordinal logistic regression model was applicable for this analysis. The results are shown in **Table 7**. IL-1β (OR = 1.408, 95% CI: 1.134-1.749, *P* = 0.001), IL-6 (OR = 1.597, 95% CI: 1.177-2.168, *P* = 0.002), IL-8 (OR = 1.224, 95% CI: 1.094-1.366, *P* < 0.001), and TNF-α (OR = 1.595, 95% CI: 1.177-2.161, *P* = 0.002) are independent risk factors for the aggravation of corneal injury severity.

**Table 7:** Results of ordered logistic regression analysis .

Tear cytokines	Univariate analysis			Multivariate analysis		
	OR	95% CI	*P*	OR	95% CI	*P*
IL-1β	1.142	1.096-1.190	<0.001	1.408	1.134-1.749	0.001
IL-2	1.287	1.186-1.396	<0.001	1.092	0.855-1.397	0.485
IL-4	0.986	0.954-1.020	0.417	–	–	–
IL-6	1.347	1.243-1.460	<0.001	1.597	1.177-2.168	0.002
IL-8	1.155	1.106-1.206	<0.001	1.224	1.094-1.366	<0.001
IL-10	0.985	0.962-1.009	0.218	–	–	–
TNF-α	1.387	1.275-1.509	<0.001	1.595	1.177-2.161	0.002
IL-17A	3.152	0.931-10.667	0.065	–	–	–
IFN-γ	1.941	0.779-4.837	0.155	–	–	–
TGF-β1	1.009	0.982-1.037	0.512	–	–	–
VEGF	0.991	0.953-1.031	0.667	–	–	–
MMP-9	3.721	0.943-14.690	0.060	–	–	–

IL-1β, interleukin-1 beta; IL-2, interleukin-2; IL-4, interleukin-4; IL-6, interleukin-6; IL-8, interleukin-8; IL-10, interleukin-10; TNF-α, tumor necrosis factor-alpha; IL-17A, interleukin-17A; IFN-γ, interferon-gamma; TGF-β1, transforming growth factor-beta 1; VEGF, vascular endothelial growth factor; MMP-9, matrix metalloproteinase-9.

## Discussion

Corneal injury is a common ocular surface lesion in clinical practice, and its severity directly affects the visual function and quality of life of patients (24). Moreover, the inflammatory response plays a central regulatory role in the occurrence, development and repair of corneal injury (25, 26). Tear fluid is a crucial component of the ocular surface microenvironment, and the levels of cytokines it contains can directly reflect the severity of corneal inflammation and the status of inflammatory activation (27, 28). However, there is a lack of clear understanding of the key inflammatory regulatory factors and early predictive indicators that determine the severity of corneal injury, which hinders the formulation of precise intervention strategies. Based on this, our study conducted systematically analyzed the tear cytokine profiles of patients with mild, moderate and severe corneal injuries. The roles and predictive values of key cytokines in the progression of corneal injuries were revealed using logistic regression and ROC curve analysis, providing experimental evidence for the early identification of high-risk patients and the development of personalized intervention strategies in clinical practice.

DES is a common ocular surface disorder, characterized by persistent ocular surface inflammation and impaired tear film stability. Chronic inflammation and high osmotic pressure can cause oxidative stress and other reactions in the cornea, thereby damaging the stability of the tear film and the integrity of the epithelium, ultimately leading to occurrence of DES (11). Previous research has verified that the tear cytokine profile in DES patients is significantly abnormal compared with that in healthy individuals. The expression levels of various factors such as IL-1β, IL-2, IL-4, IL-6, IL-17A, IFN-γ, TNF-α, and MMP-9 all show a significant upward trend (29). In this study, the overall comparison of the three severity groups revealed that the levels of cytokines including IL-1β, IL-6, IL-8, TNF-α, IL-17A, IFN-γ and MMP-9 gradually increased as the corneal injury worsened. Pairwise comparisons further confirmed that the levels of these cytokines were significantly higher in the moderate group than in the mild group, and they continued to rise in the severe group. Horwitz et al. detected damaged corneal tissue and found that the expression levels of IL-1β, IL-6, IL-8, TNF-α, and IL-17A in the damaged corneal tissue, as well as the levels of IL-8 and MCP-1 in the limbal area, were significantly higher than those in the normal un-injured eye (30). Den et al.’s research also confirmed that in the untreated corneal injury model, IL-1β, IL-6, and IL-8 showed a significant upward trend within 7 days after injury, further supporting the core role of pro-inflammatory factors in the pathological process of corneal injury (31).

The above research evidence all points to the close association between inflammatory factors such as IL-1β, IL-6, IL-8, and TNF-α and corneal injury. However, whether these factors have independent regulatory effects during the progression of corneal injury still needs further verification. Logistic regression analysis showed that IL-1β, IL-6, IL-8, and TNF-α were independent risk factors during the progression from mild disease to moderate disease. In contrast, IL-1β, IL-6, and TNF-α continued to be independent risk factors during the progression from moderate disease to severe disease. Furthermore, the ordered logistic regression analysis confirmed that IL-1β, IL-6, TNF-α and IL-8 were independent risk factors for the aggravation of the severity of corneal injury at all levels. Zhang et al. discovered that the corneal injury in DES patients is closely related to the inflammatory response mediated by IL-1β, IL-6, and TNF-α (32). Na and Chen’s studies also found that IL-6 and IL-8 are key associated factors for corneal injury related to dry eye (33, 34). Wang et al. discovered that the abnormal increase of IL-1β in the tears of patients with DES would exacerbate the inflammatory response of the corneal epithelium, disrupt the stability of the tear film, and ultimately lead to symptoms related to dry eye and corneal dysfunction (35). Zhou et al. found that inhibiting TNF-α could alleviate corneal stromal stem cell damage in eye injury (36).We conducted ROC curve analysis on the significant inflammatory factors from the logistic regression analysis results. In the prediction of mild to moderate progression, the AUC of the combined model of IL-1β, IL-6, IL-8, and TNF-α reached 0.865, with a sensitivity of 80.49% and a specificity of 89.80%, which was significantly higher than each single factor. Bootstrap internal validation confirmed the stability of this model. In the prediction of moderate to severe progression, the AUC of the combined model of IL-1β, IL-6, and IL-6 further increased to 0.885, with a sensitivity of 82.35% and a specificity of 87.80%, demonstrating better diagnostic performance. Bootstrap validation also supported the robustness of this model. The results showed that each independent risk factor detected alone had certain predictive value for the progression of corneal injury, but the combined detection had significantly better efficacy than a single factor.

This study still has some limitations. Firstly, the sample size included in this study is relatively small. In the future, multi-center large-sample studies are needed to verify the generalizability of the conclusions. Secondly, the association between cytokines and dry eye subtypes was not analyzed. The pathological mechanisms of different subtypes may differ, and the characteristic of the cytokine spectrum of each subtype needs to be further explored.

## Conclusion

This study confirmed that the profiles of tear cytokines exhibit dynamic changes during the progression of corneal injury in DES. Inflammatory factors such as IL-1β, IL-6, IL-8, and TNF-α are significantly correlated with the severity of corneal injury and may serve as core mediators involved in the injury process. For the IL-1β + IL-6 + IL-8 + TNF-α combined model for mild-to-moderate injuries and the IL-1β + IL-6 + IL-8 comprehensive model for moderate-to-severe injuries, they respectively exhibit excellent predictive performance. These models provide reliable experimental evidence for the grading management, risk warning, and individualized intervention of corneal injury in DES, and have significant clinical significance for promoting precise diagnosis and treatment of DES. It has important clinical significance for promoting precise diagnosis and treatment of DES.

## Data Availability

The original contributions presented in the study are included in the article/supplementary material. Further inquiries can be directed to the corresponding author.
